# Supplementary material for: Single-cell analysis of psoriasis resolution demonstrates an inflammatory fibroblast state targeted by IL-23 blockade
Source: Nat Commun. 2024 Jan 30;15:913. doi: 10.1038/s41467-024-44994-w (PMC10828502; doi:10.1038/s41467-024-44994-w)
Supplement: Supplementary file 1 — Supplementary Information [file 41467_2024_44994_MOESM1_ESM.pdf]

Supplementary Figures

A

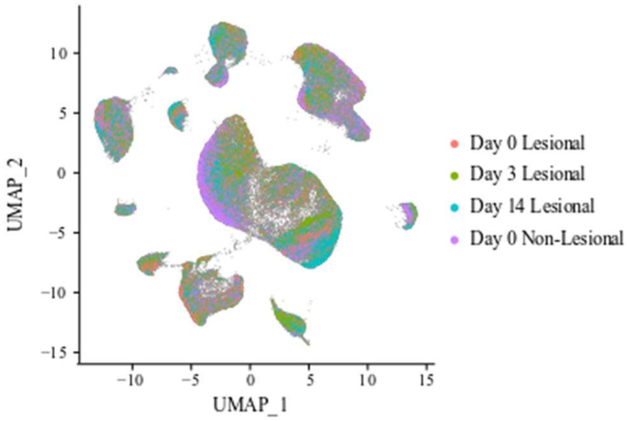

B

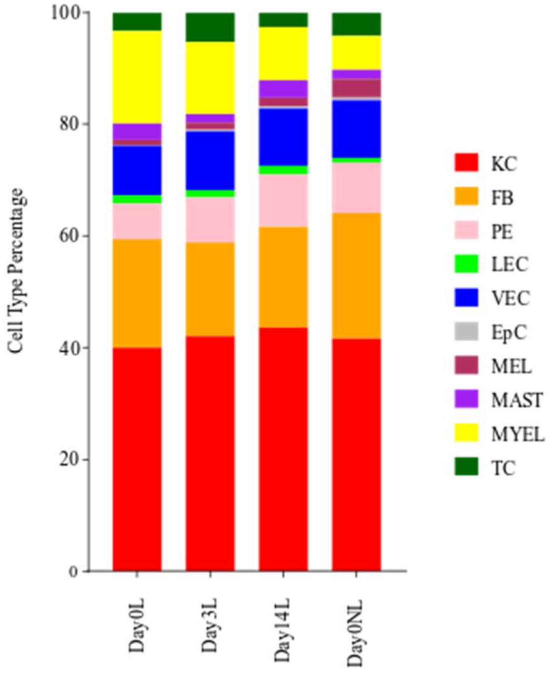

Supplementary Figure 1: Clustering and abundance of cell populations detected in treated and untreated skin. (**A**) Unifold Manifold Approximation and Projection (UMAP) of 164,553 single cells showing that cluster membership is not influenced by sample type (lesional vs non-lesional skin) or study time point (n=5 patients). (**B**) Stacked bar chart showing the abundance of the various cell

populations in the four sample groups. KC, keratinocytes; FB, fibroblasts; PE, pericytes; LEC, lymphatic endothelial cells; VEC, vascular endothelial cells; EpC, epithelial cells; MEL, melanocytes; MAST, mast cells; MYEL, myeloid cells; TC, T cells; L, lesional skin; NL, non-lesional skin.

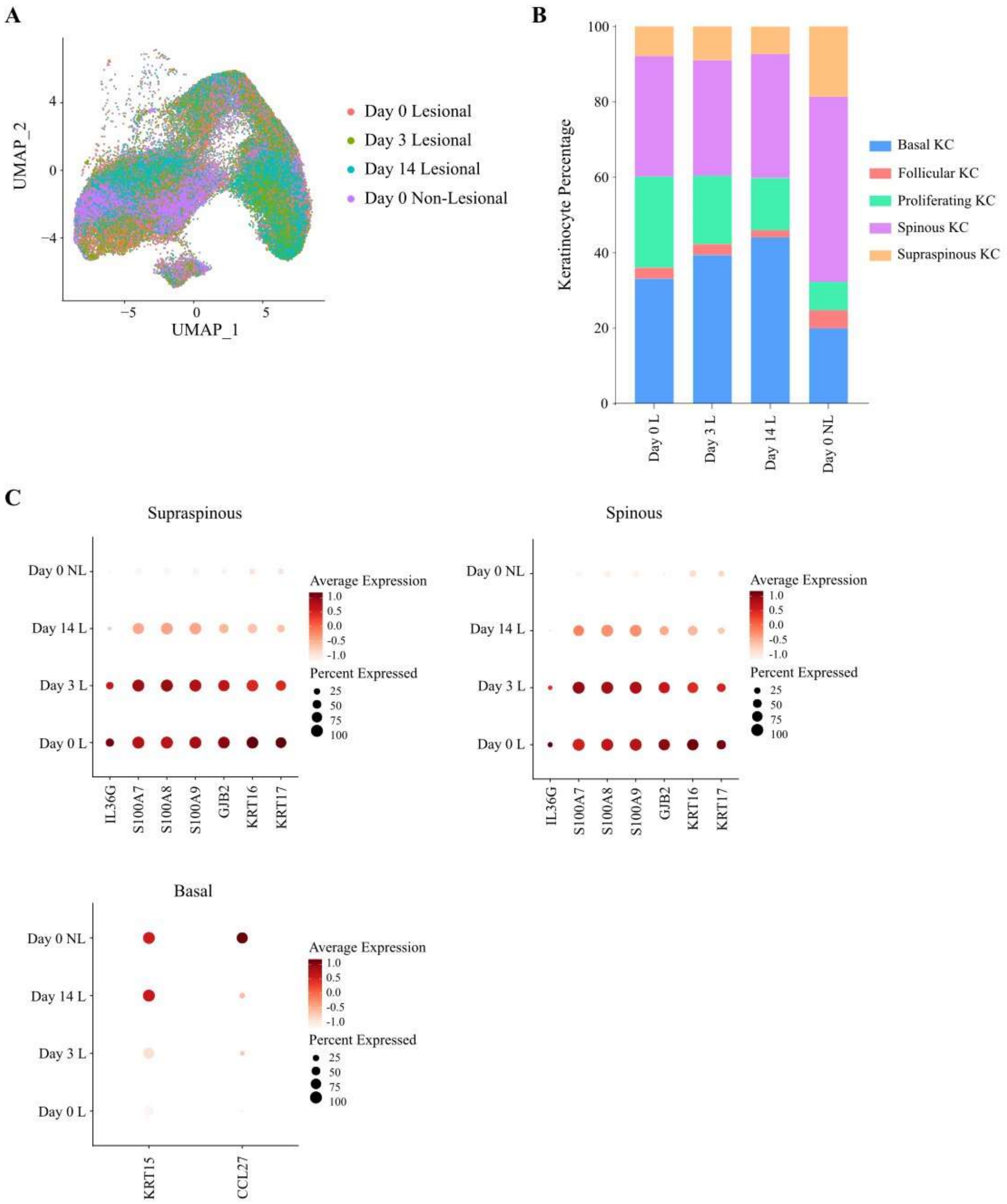

Supplementary Figure 2: Clustering and abundance of keratinocyte populations detected in treated and untreated skin. (**A**) UMAP of 68,695 keratinocytes showing that cluster membership is not influenced by sample type (lesional vs non-lesional skin) or study time point (n=5 patients). (**B**) Stacked bar chart showing the abundance of the various keratinocyte populations in the four sample groups. (**C**) Dot plots showing treatment-induced gene expression changes within keratinocyte populations. KC, keratinocytes; L, lesional skin; NL, non-lesional skin.

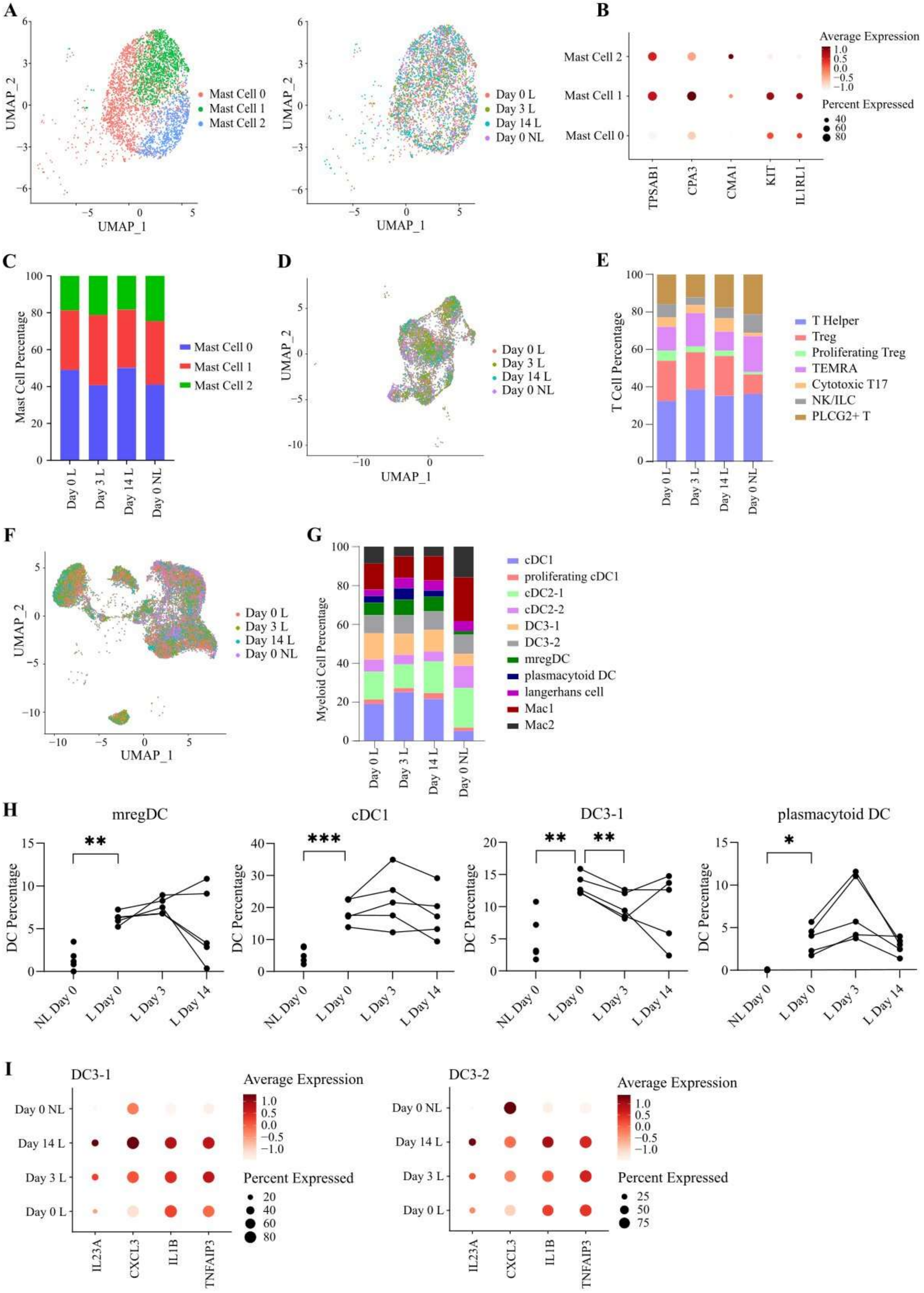
A
UMAP_2
UMAP_1
Mast Cell 0
Mast Cell 1
Mast Cell 2
Day 0 L
Day 3 L
Day 14 L
Day 0 NL
B
Average Expression
Percent Expressed
Mast Cell 2
Mast Cell 1
Mast Cell 0
TPSAB1
CPA3
CMA1
KIT
IL1RL1
C
Mast Cell Percentage
D
E
T Cell Percentage
T Helper
Treg
Proliferating Treg
TEMRA
Cytotoxic T17
NK/ILC
PLCG2+ T
F
G
Myeloid Cell Percentage
cDC1
proliferating cDC1
cDC2-1
cDC2-2
DC3-1
DC3-2
mregDC
plasmacytoid DC
langerhans cell
Mac1
Mac2
H
mregDC
cDC1
DC3-1
plasmacytoid DC
DC Percentage
NL Day 0
L Day 0
L Day 3
L Day 14
I
DC3-1
DC3-2
Day 0 NL
Day 14 L
Day 3 L
Day 0 L
IL23A
CXCL3
IL1B
TNFAIP3

Supplementary Figure 3: Clustering and abundance of immune populations detected in treated and untreated skin. (**A**) UMAP of 3,755 mast cells forming three distinct sub-clusters. Cells are coloured by sub-cluster (left) or sample group (right) (n=5 patients). (**B**) Dot plot showing the expression of marker genes in the three sub-clusters. (**C**) Stacked bar chart showing the abundance of the various mast cell populations in the four sample groups. (**D**) UMAP of 6,263 T cells showing that cluster membership is not influenced by sample type (lesional vs non-lesional skin) or study time point. (**E**) Stacked bar chart showing the abundance of the various T cell populations in the four sample groups. (**F**) UMAP of 18,544 myeloid cells showing that cluster membership is not influenced by sample type (lesional vs non-lesional skin) or study time point. (**G**) Stacked bar chart showing the abundance of the various myeloid populations in the four sample groups. (**H**) Plot showing the abundance of myeloid populations at different time points. Every line represents a patient. *P<0.05, *P<0.01, *P<0.001 (repeated measures ANOVA with Dunnett's post-test). (**I**) Dot plots showing treatment-induced gene expression changes within DC3-1 (left) and DC3-2 (right) cells. Treg, regulatory T cell; TEMRA, terminally differentiated effector memory T cell; NK, natural killer cell; ILC, innate lymphoid cell; DC, dendritic cell; Mac, macrophage; L, lesional skin; NL, non-lesional skin.

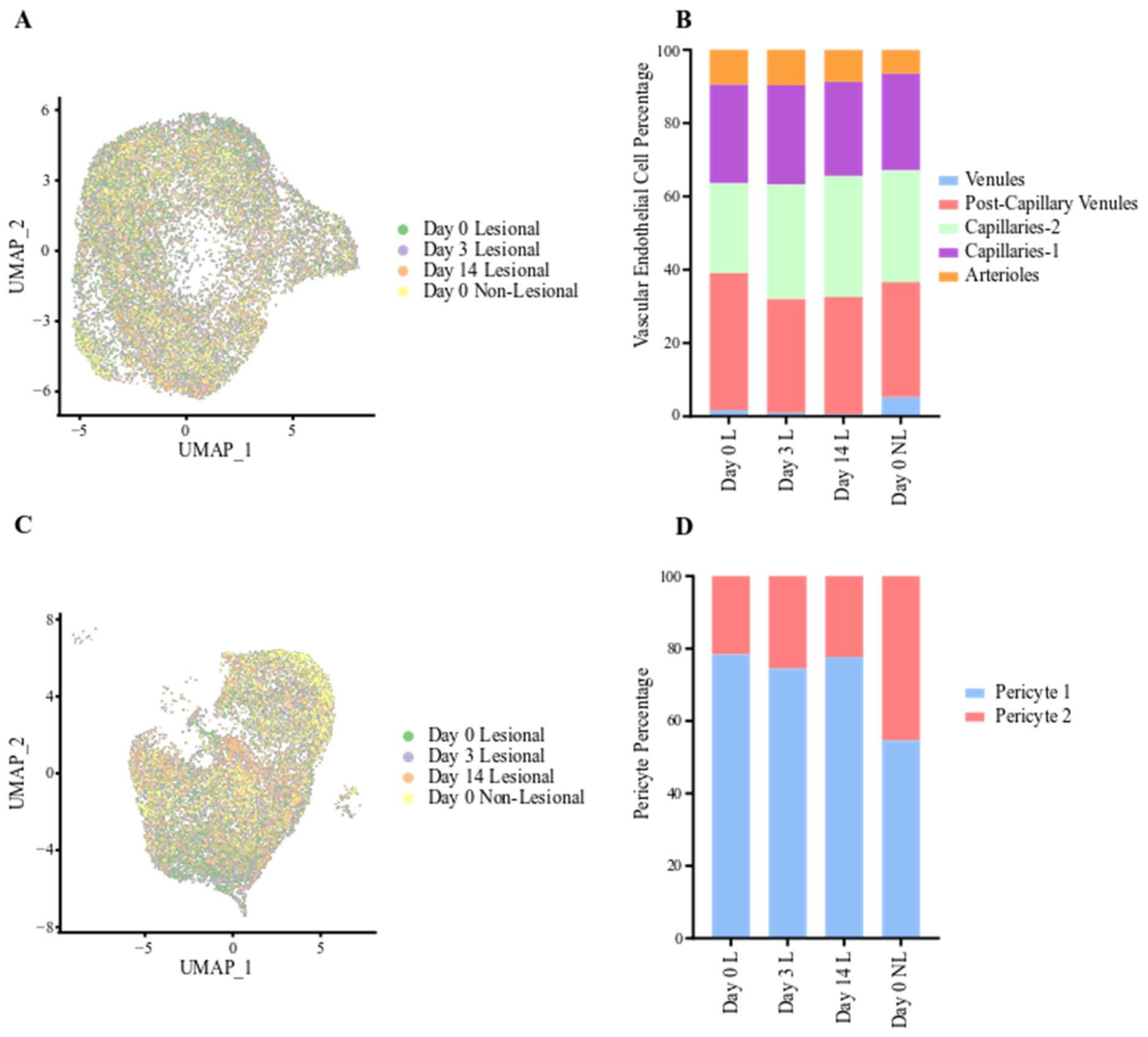

Supplementary Figure 4: **Clustering and abundance of vascular endothelial cells and pericytes in treated and untreated skin**. (**A**) UMAP of 16,420 vascular endothelial cells showing that cluster membership is not influenced by sample type (lesional vs non-lesional skin) or study time point (n=5 patients). (**B**) Stacked bar chart showing the abundance of the various vascular endothelial cell populations in the four sample groups. (**C**) UMAP of 13,507 pericytes showing that cluster membership is not influenced by sample type (lesional vs non-lesional skin) or study time point. (**D**) Stacked bar chart showing the abundance of the various pericyte populations in the four sample groups.

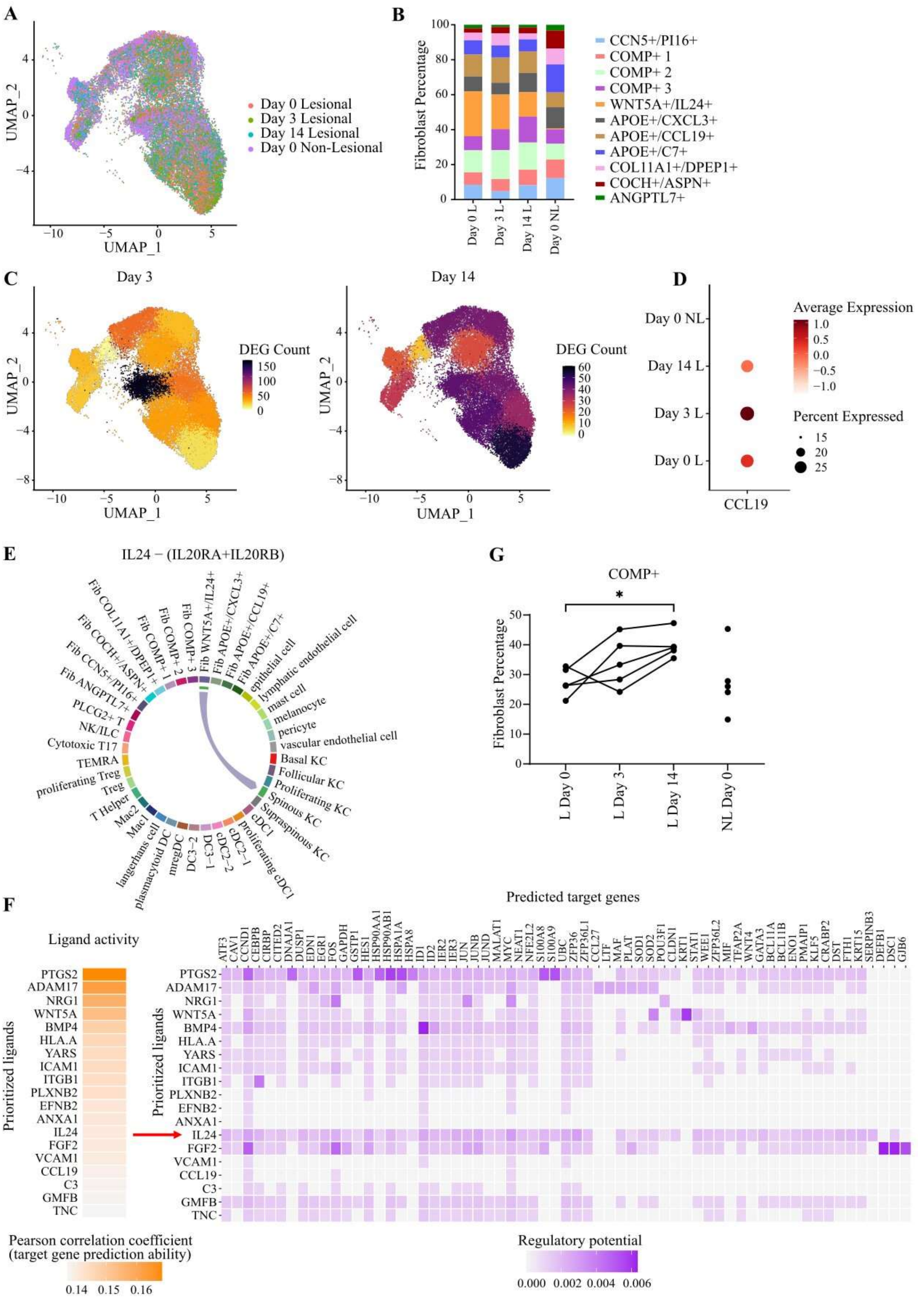
A
B
C
D
E
F
G
UMAP_1
UMAP_2
Day 0 Lesional
Day 3 Lesional
Day 14 Lesional
Day 0 Non-Lesional
Fibroblast Percentage
CCN5+/PI16+
COMP+ 1
COMP+ 2
COMP+ 3
WNT5A+/IL24+
APOE+/CXCL3+
APOE+/CCL19+
APOE+/C7+
COL11A1+/DPEP1+
COCH+/ASPN+
ANGPTL7+
Day 0 L
Day 3 L
Day 14 L
Day 0 NL
Day 3
Day 14
DEG Count
Average Expression
Percent Expressed
CCL19
IL24 − (IL20RA+IL20RB)
COMP+
L Day 0
L Day 3
L Day 14
NL Day 0
Predicted target genes
Ligand activity
Prioritized ligands
Pearson correlation coefficient
(target gene prediction ability)
Regulatory potential

Supplementary Figure 5: **Clustering and abundance of fibroblast populations detected in treated and untreated skin.** (**A**) UMAP of 31,765 fibroblasts showing that cluster membership is not influenced by sample type (lesional vs non-lesional skin) or study time point (n=5 patients). (**B**) Stacked bar chart showing the abundance of the various fibroblast populations in the four sample groups. (**C**) UMAP visualization of fibroblasts from lesional skin, showing the number of DEG observed in each cluster, after 3 (left) and 14 (right) days of treatment. (**D**) Dot plot showing the expression of *CCL19* in fibroblasts, following treatment with risankizumab. (**E**) Inferred interaction between IL-24 produced by *WNT5A*+/*IL24*+ fibroblasts and its receptor (encoded by *IL20RA* and *IL20RB*) on spinous keratinocytes. (**F**) NicheNet ligand activity prediction for the communication between *WNT5A+/IL24+* fibroblasts and spinous keratinocytes. Left: potential ligands expressed by *WNT5A+/IL24+* fibroblasts ranked according to their ability (Pearson correlation coefficient) to predict the gene expression changes observed between day 0 and day 14. Right: matrix showing the potential targets of the top ranked ligands and their regulatory potential in spinous keratinocytes. (**G**) Plot showing the abundance of COMP+ fibroblasts at different time points. Every line represents a patient. Fib, fibroblasts; L, lesional skin; NL, non-lesional skin; *P<0.05 (repeated measures ANOVA with Dunnett's post-test).

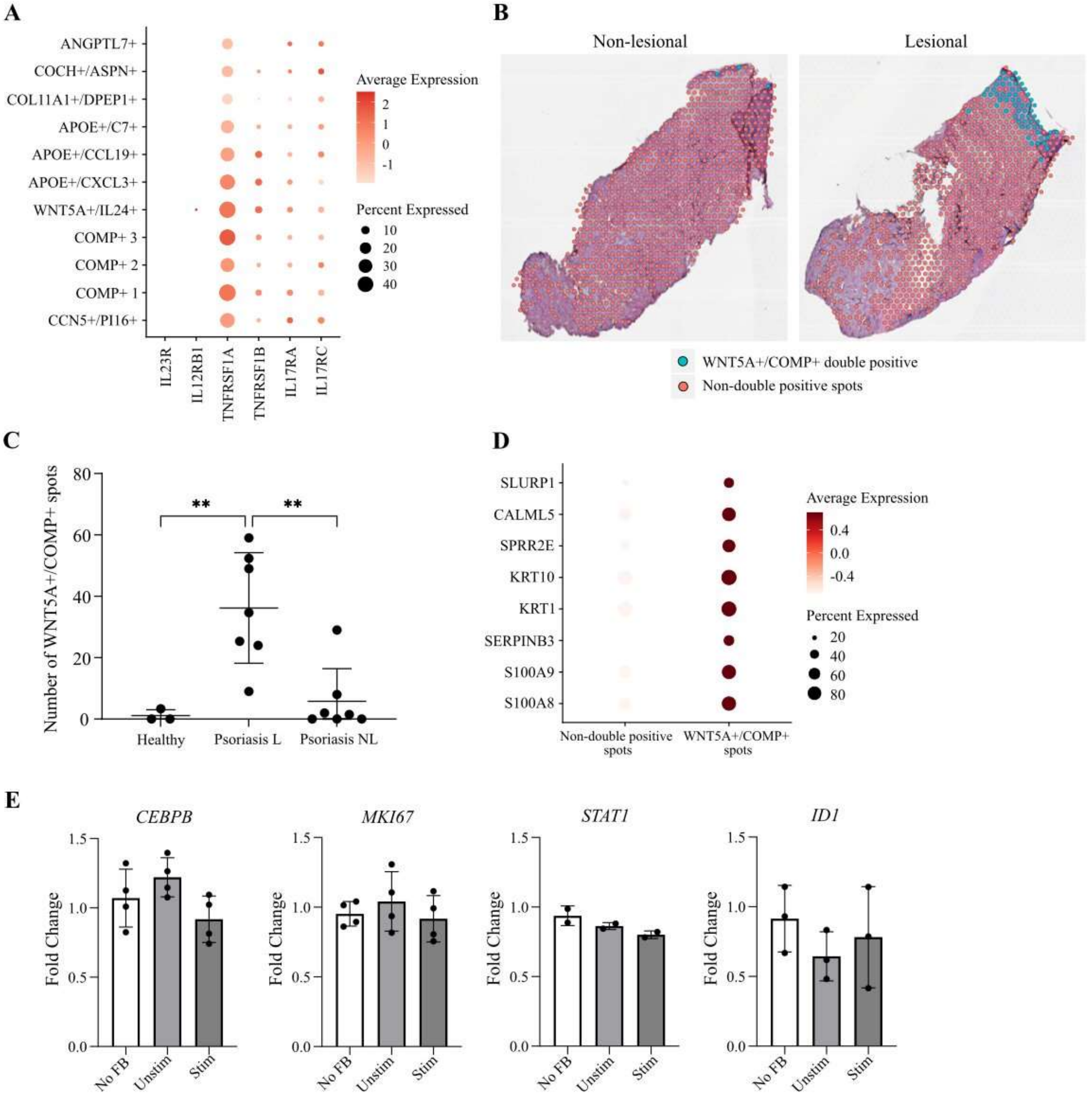

Supplementary Figure 6: **Spatial localization of *WNT5A+/IL24+* fibroblasts and interacting partners.** (**A**) Dot plot showing the expression of immune receptor genes in the various fibroblast populations. (**B**) Representative spatial plots from non-lesional and lesional psoriasis skin showing the localization of *WNT5A+/COMP+* spots. (**C**) Number of *WNT5A+/COMP+* spots across healthy (n=3), psoriasis lesional (n=7) and psoriasis non-lesional skin (n=7) conditions; ***P*<0.01 (ANOVA with Dunnett's post-test). (**D**) Dot plot showing the expression of spinous/supra-spinous keratinocyte markers in *WNT5A+/COMP+* spots vs all other spots. (**E**) Real-time PCR analysis of inflammatory markers in human primary keratinocytes cultured with unconditioned medium (no FB grown), supernatants from IL-17A/TNF stimulated fibroblasts

(Stim) or control medium (supernatant from unstimulated fibroblasts, Unstim), n=4 biologically independent samples for *CEBPB* and *MKI67*, n=3 for *ID1* and n=2 for *STAT1*; (ANOVA with Dunnett's post-test); data are mean (SD). L, lesional skin; NL, non-lesional skin.

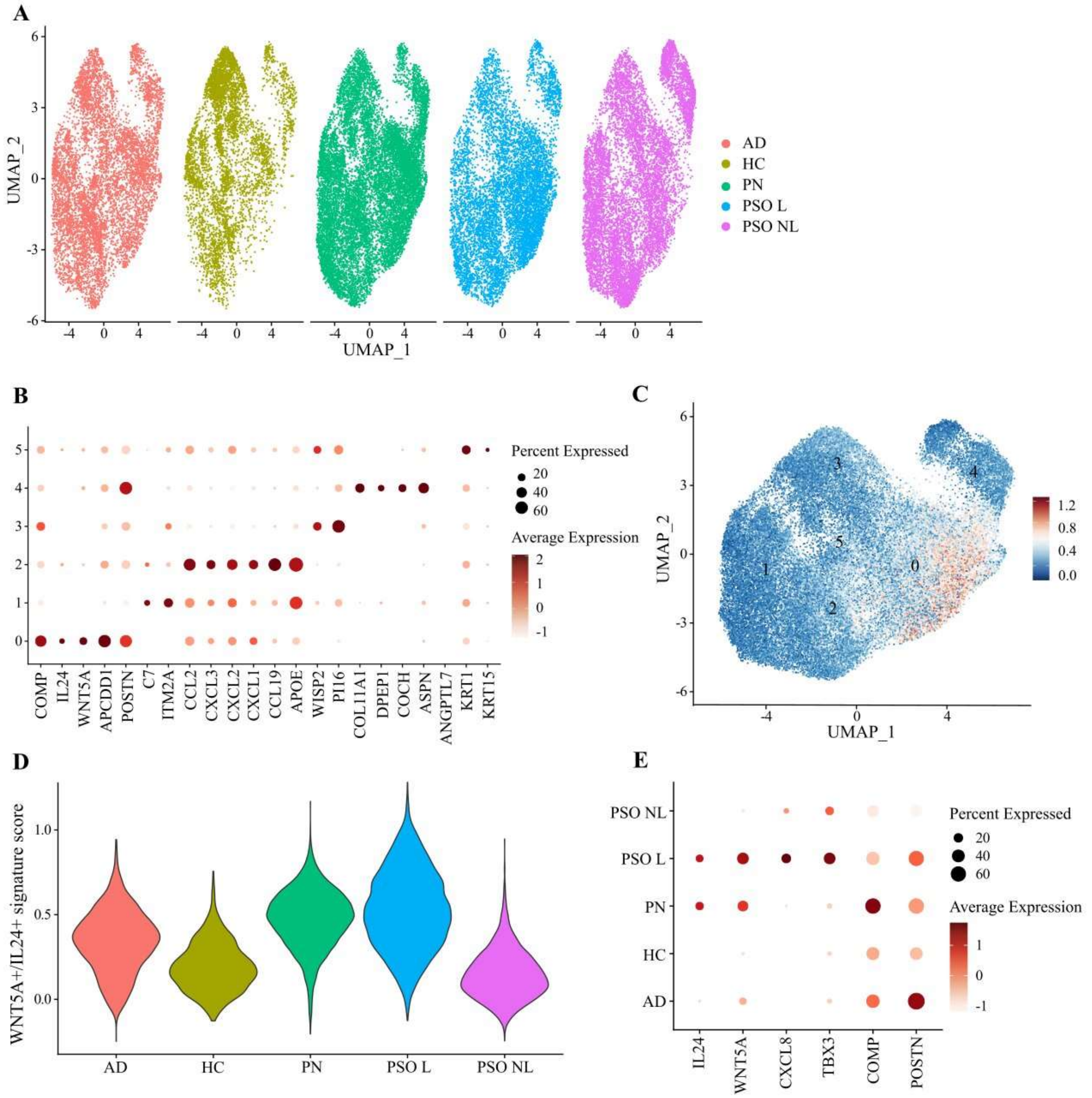

Supplementary Figure 7: **Analysis of *WNT5A+/IL24+* fibroblasts across inflammatory skin diseases.** (**A**) UMAP of fibroblasts from publicly available scRNA-seq datasets of healthy (n=4), prurigo nodularis (n=7) and atopic dermatitis (n=5) skin, following integration with our day 0 psoriasis data (n=5). Cells are coloured by sample group. (**B**) Dot plot showing the expression of marker genes used for the annotation of fibroblast sub-clusters. The *WNT5A+/IL24+* population corresponds to cluster 0 (**C**) UMAP of fibroblasts populations confirming that the *WNT5A+/IL24+* cell signature is specific to cluster 0. (**D**) Violin plot showing the *WNT5A+/IL24+* signature score across sample groups. (**E**) Dot plot showing gene expression in *WNT5A+/IL24+* fibroblasts across sample groups. PSO, psoriasis; L, lesional skin; NL, non-lesional skin; HC, healthy control; PN, prurigo nodularis; atopic dermatitis, AD.

Supplementary Tables

Supplementary Table 1: Patient demographics[1]

	Patient n.	*Mean age (SD)*	*Sex*	*Comorbidity*	*Mean baseline PASI (SD)*	*Biologic naïve*
Discovery (scRNA-seq)	5	30.1 (9.6)	5M	None	23.8 (9.9)	Yes
Validation (RNA-scope)	3	41.3 (10.1)	2M 1F	None	27.7 (11.7)	Yes

[1]All individuals were of European descent; M, male; F, female; PASI, psoriasis area and severity index.

Supplementary Table 2: scRNA-seq output summary statistics

Sample Group	*Mean n. of cells per sample (SD)*	*Mean n. of reads per cell (SD)*	*Mean n. of genes per cell (SD)*
Non-lesional skin Day 0	8970 (1325)	6967 (4427)	1900 (776)
Lesional skin Day 0	8382 (1583)	7134 (5384)	1953 (921)
Lesional skin Day 3	7906 (1522)	7138 (5552)	1886 (902)
Lesional skin Day 14	7653 (2384)	7022 (4779)	1944 (875)

Supplementary Table 3: Real-time PCR primers

Target	*Primer sequences (5' to 3')*
CEBPB	AACTCTCTGCTTCTCCCTCTG<br>AAGCCCGTAGGAACATCTTT
CXCL8	GAGAAGTTTTTGAAGAGGGCTGA<br>CTTCACTGATTCTTGGATACCACA
GAPDH[1]	CGGAGTCAACGGATTTGGTC<br>AATGAAGGGGTCATTGATGGCA
ID1	CAGTTGGAGCTGAACTCGGA<br>AACGCATGCCGCCTCG
IL24	TGTGGACTTTAGCCAGACCC<br>GGTAAAACCCAGGCAAGGGA
MKI67	CTTTGGGTGCGACTTGACGA<br>ACAACTCTTCCACTGGGACG
PKG1[1]	GCGGGTCGTTATGAGAGTCG<br>TGGGACAGCAGCCTTAATCC
S100A8	TTTCAGAAGACCTGGTGGGG<br>CAGGGAGTACTTGTGGTAGACG
S100A9	TCCTCGGCTTTGACAGAGTG<br>TGGTCTCTATGTTGCGTTCCA
SERPINB3	ACCAATGTGGTATTGCTGCCAA<br>AACTCCTGGGTGGAAAGTCAA
STAT1	TCTGTGTCTGAAGTTCACCCT<br>TCCGAGACACCTCGTCAAAC
TBX3	TGCACCTGGAGGCTAAAGAAC<br>GGAAACATTCGCCTTCCCGA
WNT5A	AGGGCTCCTACGAGAGTGCT<br>GACACCCCATGGCACTTG

[1]Housekeeping genes
