# Supplementary material for: Single-cell analysis of psoriasis resolution demonstrates an inflammatory fibroblast state targeted by IL-23 blockade
Source: Nat Commun. 2024 Jan 30;15:913. doi: 10.1038/s41467-024-44994-w (PMC10828502; doi:10.1038/s41467-024-44994-w)
Supplement: Supplementary file 3 — Reporting Summary [file 41467_2024_44994_MOESM3_ESM.pdf]

nature portfolio

Corresponding author(s): Francesca Capon, Satveer Mahil

Last updated by author(s): Dec 19, 2023

Reporting Summary

Nature Portfolio wishes to improve the reproducibility of the work that we publish. This form provides structure for consistency and transparency in reporting. For further information on Nature Portfolio policies, see our Editorial Policies and the Editorial Policy Checklist.

Statistics

For all statistical analyses, confirm that the following items are present in the figure legend, table legend, main text, or Methods section.

n/a	Confirmed	
☐	☒	The exact sample size (*n*) for each experimental group/condition, given as a discrete number and unit of measurement
☐	☒	A statement on whether measurements were taken from distinct samples or whether the same sample was measured repeatedly
☐	☒	The statistical test(s) used AND whether they are one- or two-sided<br>*Only common tests should be described solely by name; describe more complex techniques in the Methods section.*
☐	☒	A description of all covariates tested
☐	☒	A description of any assumptions or corrections, such as tests of normality and adjustment for multiple comparisons
☐	☒	A full description of the statistical parameters including central tendency (e.g. means) or other basic estimates (e.g. regression coefficient) AND variation (e.g. standard deviation) or associated estimates of uncertainty (e.g. confidence intervals)
☐	☒	For null hypothesis testing, the test statistic (e.g. *F*, *t*, *r*) with confidence intervals, effect sizes, degrees of freedom and *P* value noted<br>*Give P values as exact values whenever suitable.*
☒	☐	For Bayesian analysis, information on the choice of priors and Markov chain Monte Carlo settings
☐	☒	For hierarchical and complex designs, identification of the appropriate level for tests and full reporting of outcomes
☐	☒	Estimates of effect sizes (e.g. Cohen's *d*, Pearson's *r*), indicating how they were calculated

Our web collection on statistics for biologists contains articles on many of the points above.

Software and code

Policy information about availability of computer code

Data collection	No software was used to collect the data
Data analysis	The following software was used for data analysis (as referenced in the manuscript): Cell Ranger v6.1.1 (10X Genomics), scDblFinder package (v3.1.6), Seurat package (v4.1.0), Ingenuity Pathway Analysis package (Qiagen), igraph (v1.2.6) R Package, SCENIC (v1.3.0), CellChat (v1.4.0), NicheNet (v1.1.1), Monocle 3 (v1.3.1), CIBERSORTx, GraphPad Prism (version 9.3.0 for Windows), Harmony (v1.0.1).

For manuscripts utilizing custom algorithms or software that are central to the research but not yet described in published literature, software must be made available to editors and reviewers. We strongly encourage code deposition in a community repository (e.g. GitHub). See the Nature Portfolio guidelines for submitting code & software for further information.

Data

Policy information about availability of data

All manuscripts must include a data availability statement. This statement should provide the following information, where applicable:

- Accession codes, unique identifiers, or web links for publicly available datasets
- A description of any restrictions on data availability
- For clinical datasets or third party data, please ensure that the statement adheres to our policy

The scRNA-seq data associated generated with this manuscript in this study have been deposited in the NCBI Gene Expression Omnibus (series id:under accession code GSE228421 (https://www.ncbi.nlm.nih.gov/geo/query/acc.cgi?acc=GSE228421)). The transcriptomic datasets generated by Castillo et al., Alkon et al., Sofen et al., Krueger et al., and Cai et al., used in this study are available in the NCBI Gene Expression Omnibus under accession codes GSE202011 (https://

www.ncbi.nlm.nih.gov/geo/query/acc.cgi?acc=GSE202011; data object can be downloaded from https://zenodo.org/records/7562864), GSE222840 (https://www.ncbi.nlm.nih.gov/geo/query/acc.cgi?acc= GSE222840), GSE51440 (https://www.ncbi.nlm.nih.gov/geo/query/acc.cgi?acc= GSE51440), GSE31652 (https://www.ncbi.nlm.nih.gov/geo/query/acc.cgi?acc= GSE31652) and GSE114729 (https://www.ncbi.nlm.nih.gov/geo/query/acc.cgi?acc= GSE114729), respectively. The rest of the data needed to evaluate the conclusions of the study are present in the paper or the Supplementary Materials.

Research involving human participants, their data, or biological material

Policy information about studies with human participants or human data. See also policy information about sex, gender (identity/presentation), and sexual orientation and race, ethnicity and racism.

Reporting on sex and gender	Self-reported sex of participants has been reported in the manuscript.
Reporting on race, ethnicity, or other socially relevant groupings	All individuals were of European descent, which has been specified in the manuscript.
Population characteristics	Relevant population characteristics have been reported in the manuscript including past and current treatment, comorbidities, and disease (psoriasis) severity. All participants were biologic-naïve, adults of European descent, with a dermatologist confirmed diagnosis of severe psoriasis (PASI > 10) (full details in Supplementary Table 1). To minimize confounders, we also applied these additional inclusion criteria: no comorbidities, no systemic immune-modifying therapy for at least 4 weeks prior to risankizumab treatment, no topical steroids to the sampled area for at least 5 days prior to sampling. All biopsies were taken from site-matched areas on the lower back/buttocks.
Recruitment	All participants had a dermatologist confirmed diagnosis of severe psoriasis (PASI > 10) and were eligible for biologic therapy according to NICE criteria (CG153). Participants were recruited via the severe psoriasis clinic (routine clinical care) at St John's Institute of Dermatology, Guy's and St Thomas' NHS Foundation Trust, London, UK.
Ethics oversight	Written informed consent was obtained from all psoriasis patients, in line with approval from the London - Westminster Research Ethics Committee (REC ref 11/H0802/7). Skin samples used for establishing primary cell cultures were obtained using REC ref 14/LO/2169.

Note that full information on the approval of the study protocol must also be provided in the manuscript.

Field-specific reporting

Please select the one below that is the best fit for your research. If you are not sure, read the appropriate sections before making your selection.

[x] Life sciences [ ] Behavioural & social sciences [ ] Ecological, evolutionary & environmental sciences

For a reference copy of the document with all sections, see nature.com/documents/nr-reporting-summary-flat.pdf

Life sciences study design

All studies must disclose on these points even when the disclosure is negative.

Sample size	The sample size was determined by the number of affected individuals matching our strict inclusion criteria (biologic-naïve, adult males of European descent, with a dermatologist confirmed diagnosis of severe psoriasis (PASI > 10). To minimise confounders and maximise statistical power, we also applied these additional inclusion criteria: no comorbidities, no systemic immune-modifying therapy for at least 4 weeks prior to risankizumab treatment, no topical steroids to the sampled area for at least 5 days prior to sampling). Given the focus on early drug-induced events, sample collection was halted after 14 days of treatment.
Data exclusions	All skin biopsies obtained from patients enrolled in the study were included in the analysis. No outliers were excluded.
Replication	The key finding of a reduction in abundance of WNT5A+/IL24+ fibroblasts after treatment was replicated in-silico, by deconvolution of multiple transcriptomic datasets, and experimentally, by RNA in situ hybridization of additional skin samples (see manuscript). The number of biological replicates for the primary fibroblast/keratinocyte experiments are reported in the relevant figure legends (Figure 5 and Supplementary Figure 6). 2 technical duplicates were performed for each condition.
Randomization	Not applicable: the study was a longitudinal cohort study and not an interventional clinical trial.
Blinding	For RNA in situ hybridization experiment it was not possible for investigators to be blinded to group allocation given the distinctive appearance of psoriasis skin pre and post treatment.

Reporting for specific materials, systems and methods

We require information from authors about some types of materials, experimental systems and methods used in many studies. Here, indicate whether each material, system or method listed is relevant to your study. If you are not sure if a list item applies to your research, read the appropriate section before selecting a response.

Materials & experimental systems

n/a	Involved in the study
☐	☒ Antibodies
☐	☒ Eukaryotic cell lines
☒	☐ Palaeontology and archaeology
☒	☐ Animals and other organisms
☐	☒ Clinical data
☒	☐ Dual use research of concern
☒	☐ Plants

Methods

n/a	Involved in the study
☒	☐ ChIP-seq
☒	☐ Flow cytometry
☒	☐ MRI-based neuroimaging

Antibodies

Antibodies used

TotalSeq™ anti-human Hashtag antibodies (Biolegend, catalogue numbers 394641, 394643, 394645, 394647, Clone LNH-94; 2M2).

Validation

Validation statements are included on the Biolegend website.
https://www.biolegend.com/en-us/punchout/punchout-products/product-detail/totalseq-b0256-anti-human-hashtag-6-antibody-17766?GroupID=GROUP28
https://www.biolegend.com/en-us/punchout/punchout-products/product-detail/totalseq-b0257-anti-human-hashtag-7-antibody-17767
https://www.biolegend.com/en-us/punchout/punchout-products/product-detail/totalseq-b0258-anti-human-hashtag-8-antibody-17768
https://www.biolegend.com/en-us/punchout/punchout-products/product-detail/totalseq-b0259-anti-human-hashtag-9-antibody-17769
'TotalSeq™ anti-human Hashtag reagents are designed to label most human cells, using a combination of two clones that recognize CD298 and β2 microglobulin, respectively. The antibodies are conjugated to the same oligonucleotide, pre-mixed to be used following an optimized protocol.'

Eukaryotic cell lines

Policy information about cell lines and Sex and Gender in Research

Cell line source(s)

Primary fibroblasts and keratinocytes used in the study were obtained from healthy donors of both sexes.

Authentication

Given these were primary cell lines, they were not authenticated. They were, however, regularly examined under a microscope and consistently displayed the relevant keratinocyte or fibroblast morphology.

Mycoplasma contamination

Not all cell lines were regularly tested for mycoplasma contamination.

Commonly misidentified lines
(See ICLAC register)

Name any commonly misidentified cell lines used in the study and provide a rationale for their use.

Clinical data

Policy information about clinical studies
All manuscripts should comply with the ICMJEguidelines for publication of clinical research and a completedCONSORT checklist must be included with all submissions.

Clinical trial registration

The study was approved by the London - Westminster Research Ethics Committee (REC ref 11/H0802/7)

Study protocol

The study design and protocol is specified in the methods section of the manuscript.

Data collection

Patients, clinical data and samples were collected at St John's Institute of Dermatology, Guy's and St Thomas' NHS Foundation Trust, London, UK (Sept 2020 - July 2022).

Outcomes

The gold standard clinical outcome measure in psoriasis was used (psoriasis area and severity index, PASI).
